# Study on the hydrothermal coupling characteristics of polyurethane insulation boards slope protection structure incorporating phase change effect

**DOI:** 10.1038/s41598-021-97561-4

**Published:** 2021-09-14

**Authors:** Hailiang Liu, Donghe Ma, Changming Wang, Xiaoyang Liu, Di Wu, Bailong Li, Kaleem Ullah Jan Khan

**Affiliations:** 1 College of Construction Engineering, Jilin University, Changchun, 130012 China; 2 China Water Northeastern Investigation, Design and Research Co., Ltd., Changchun, 130061 China

**Keywords:** Civil engineering, Structural materials

## Abstract

The canals are essential for agricultural irrigation, shipping and industry as important hydraulic infrastructure. In the seasonal freeze regions, the water conveyance canals are damaged due to the effects of freeze–thaw cycles. The freeze depth of soil in the water transfer canal varies considerably due to changes in temperature and water content. This paper compared the relationship of freeze depth, temperature and water content by field tests and numerical calculation methods by incorporating phase change. The results from present study showed that the decrease in temperature causes the water in the soil to freeze, the ice front migrated downwards, and the water in soil below ice front gradually migrated towards the ice front resulting in a large difference in water content of the soil before and after freezing. The Polyurethane insulation board + Concrete board slope structure (PC) as an insulation slope structure was proposed in this paper to mitigate the effect of freezing and thawing on the water conveyance canals. The freeze depth decreased significantly under the protective effect. In addition, this paper compared the anti-frost effect of different thicknesses of polyurethane insulation boards, and the results provided a reference for the anti-frost design of water conveyance canals.

## Introduction

**Figure 1:**
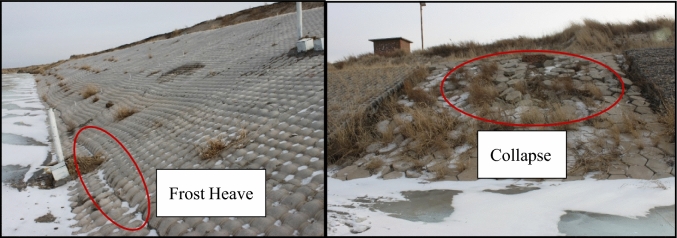
Frost damage to canals.

Frozen soil is widespread across the world, covering a total area of about 23% of the land area^1^. Different types of frozen soil cover the territory of China in which the total area of seasonally frozen soil is about 4.76 × 10^6^ km^2^ accounting for about 49.6% of China’s territory^2^. Human engineering activities inevitably conduct in seasonal freeze regions as the society develops. For example, to solve the drought problems in northern seasonal freeze regions, water conveyance canals are usually built to transfer water from water-rich areas to arid areas. The soil in seasonal freeze regions shows the characteristics of freezing and thawing under the influence of temperature. The water conveyance canals built in the seasonal freeze regions are damaged by seepage and freeze–thaw as frost heave, hollow and collapse damage (Fig. 1)^3^. The frost damage to canals has become a shackle for the safe and efficient operation of water transfer projects and economic development for arid and cold regions where water resources are extremely scarce^4–7^.

Damage to canals in the seasonal freeze regions is influenced by the environmental factors (solar radiation, air thermal convection, precipitation, evaporation, etc.), the properties of soil (permeability, water content, gradation, pore space, etc.), the groundwater table and the form of canals (section form and lining structure, etc.)^3^. The study of soil freeze can be traced back to the formulation of first and second freeze theories^8–11^. However, the theories of freeze in this period are not further developed due to constraints of experimental conditions and computational efficiency. Nevertheless, the development of two theories of freeze has contributed significantly to the subsequent researches on freeze. Since 1980, technological development has made it possible for scholars from various countries to conduct experimental studies on freeze, thus providing great convenience to study the interaction between temperature and water in soils^12,13^. Kunio and Yurie established the relationship between the permeability coefficient, temperature and ice content of the soil by unsaturated soil permeability tests^14^. In recent years, numerical methods have been widely used in solving freeze issues with the development of computing technology. Numerical calculations make it possible to simulate changes in temperature and water in the soil, providing a reference for actual projects^15–17^. Li et al. studied the mechanism of freeze in a water conveyance canal by numerical simulation and their results showed that the soil frost damage was caused by freezing of water in soil^18^. In addition, numerical analysis was equally effective in predicting the damage mechanism of lining structures, which provided a basis for the design of concrete lining protection^19^. In addition to theoretical studies and numerical simulations, the physical models are also important in the study of freezing effect of soils^15–17^. Li et al. conducted a soil bag frost protection test to investigate the effect of soil bags, their findings showed that soil bags were able to inhibit the migration of water significantly^20^.

The aforementioned research focuses on the factors influencing the development of freeze in water conveyance canal projects in seasonal freeze regions, as well as the variation characteristics and preventative measures based on theory, model tests and numerical simulations. Although numerical calculations, as well as laboratory tests, have achieved numerous achievements in the study of hydrothermal characteristics of water conveyance canals, the accuracy of the results is questioned at times due to various assumptions and boundary conditions. The physical model developed in the laboratory is unable to fulfil the temporal and dimensional effects that affect the freeze in the canals. As for the laboratory model tests, although they provide a more realistic reflection of the effects of temperature and water on freeze, using just one method (laboratory tests or numerical simulations) is inadequate in determining the freezing characteristics of the canals.

The present study examined the freezing behavior and anti-frost effects of canals of Hada Mountain Water Conservancy Project to provide information that ensures the stability and operational safety of water conveyance canals in such areas. This method solved the limitations of laboratory model tests that cannot meet the time and space scales of actual engineering. The general change law of the freezing behavior of water conveyance canals was also revealed. Finally, the numerical simulation which adequately considered the effects of ice-water phase changes was proposed to provide the important technical guidance for freeze prevention and the optimization design of anti-frost structures for water conveyance canals in seasonal freeze regions.

## Study area

**Figure 2:**
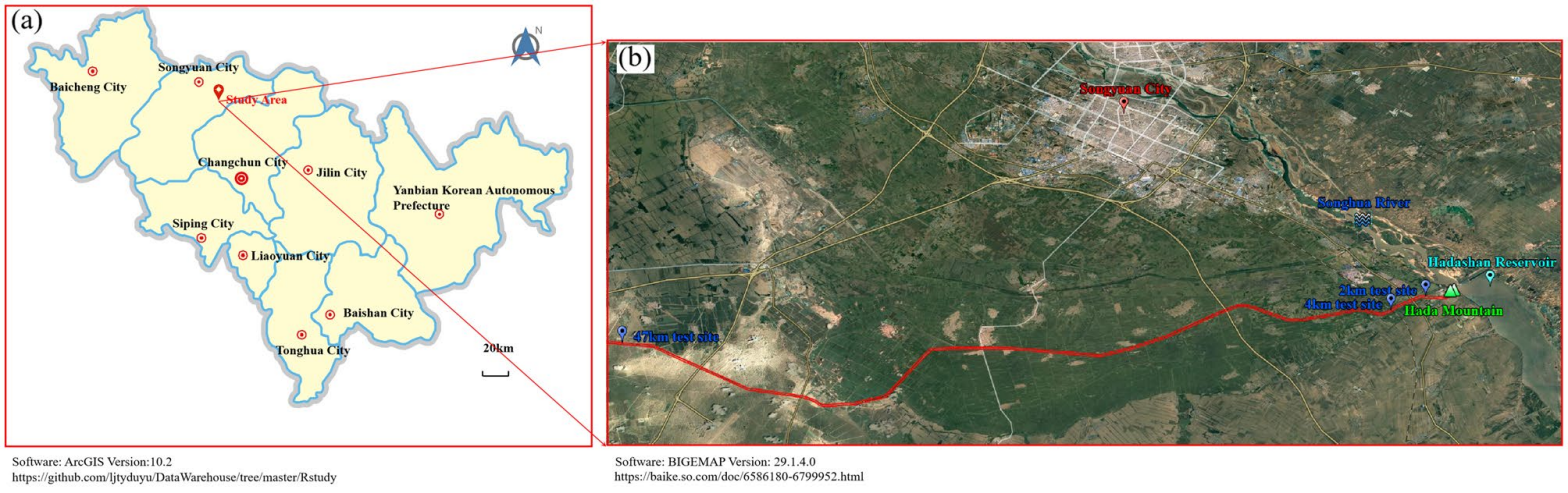
Detailed overview of the main canal of Hada Mountain Water Conservancy Project ((**a**) Geographical location map of the study area; (**b**) Satellite map of the study area).

The Hada mountain water conservancy project is located on the second mainstream of the Songhua River about 20 km from the southeast of Songyuan City, Jilin Province, China (Fig. 2a,b).

According to the statistics of meteorological data provided by Songyuan City Meteorological Service from 1971 to 2012 (41 years), the average freeze period is 123 days per year with the longest and shortest frozen periods as 146 days and 102 days respectively. Freezing has occurred from 22 October to 29 November and thawing has begun from 28 February to 31 March. The important index of coldness in a freeze–thaw cycle is the Freezing Index, which is a cumulative of negative daily average temperature (°C) during a freezing period and can be calculated based on Eq. (1).

1 $$ I_{{\text{f}}} = \mathop \smallint \limits_{{t_{0} }}^{{t_{1} }} |T|{\text{d}}t , T < 0\, ^\circ {\text{C}}, $$

where: *I*_f_ is the Freezing Index (°C·d); *t*_0_, *t*_1_ are the first and last day of the year when the temperature is below 0 °C (d); and *T* is the average daily temperature which is negative (°C).

**Figure 3:**
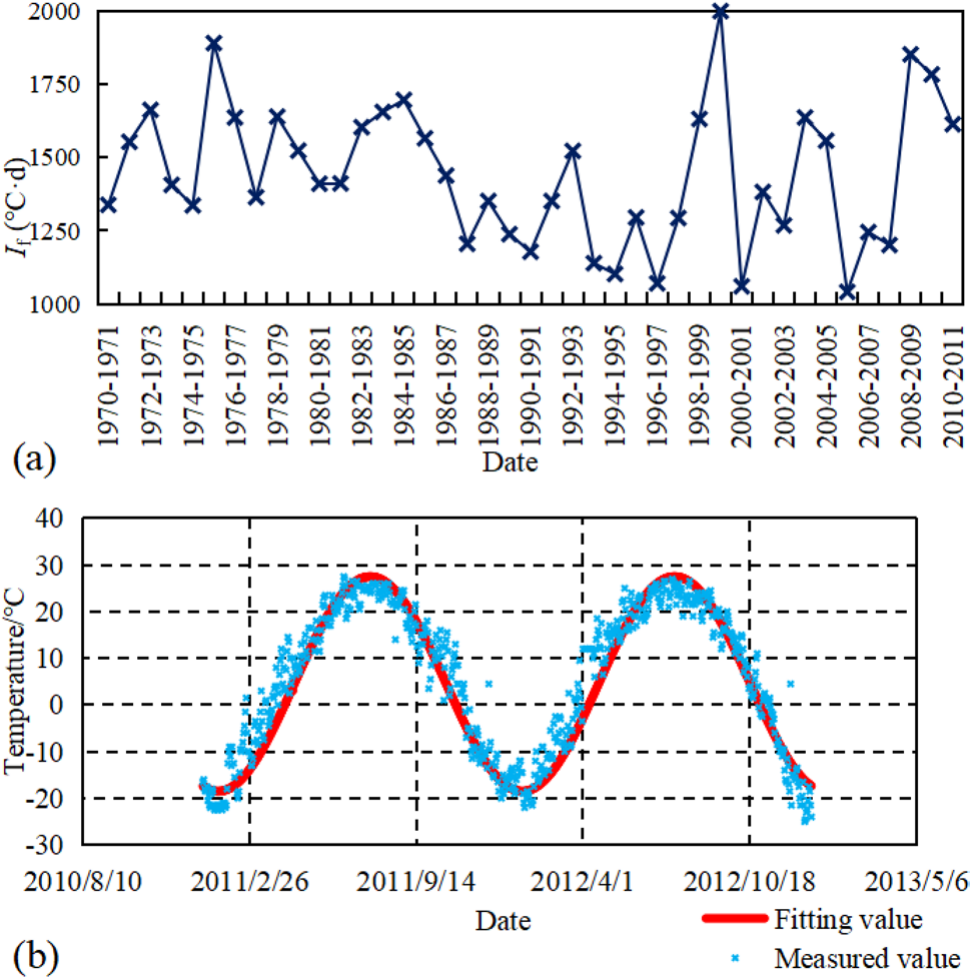
Temperature characteristics ((**a**) Freezing index curve, (**b**) daily average temperature from 2011 to 2012).

During 1971–2011, the *I*_f_ of Songyuan City is shown in Fig. 3a with a maximum *I*_f_ of 1999 °C·d, a minimum *I*_f_ of 1041 °C·d and an average *I*_f_ of 1443 °C·d. According to the temperature data in Songyuan City (Fig. 3b), the *I*_f_ for 2011–2012 is calculated as 1613 °C·d by Eq. (1). The degree of coldness in 2011–2012 is moderate according to previous *I*_f_. The cosine function was fitted to the measured average daily temperature as shown in Eq. (2) ^21^.

2 $$ T =  \overline{\text{{T}}} + {\text{A }}\cos \left( {\frac{{{\text{2}}}}\pi {\text{t}} {{{365}}} + {\uppi  }\varphi } \right){,} $$

where: *T* is the ground temperature (°C); *t* is the time (d); ${\overline{\text{T}}}$ is the average annual ground temperature (°C), $\overline{\text{{T} = 4}}{\text{.5}} ^\circ C$; A is the annual amplitude of ground temperature, A = 23 °C; *φ* the initial phase, *φ* = 0.9.

**Figure 4:**
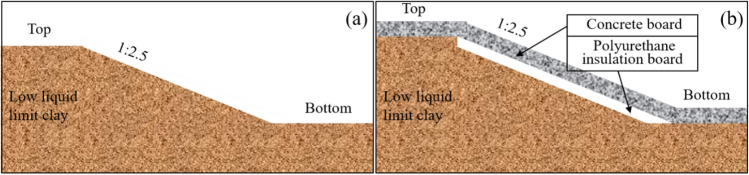
Diagram of slopes and protection scheme ((**a**) Soil slope, (**b**) PC).

The main canal length of the Hada mountain water conservancy project is 95.93 km. The canal cross-section is trapezoidal. The frost damage to the water conveyance canal under low temperature conditions is a serious problem, which threatens the safety of the project. The Polyurethane insulation board + Concrete board slope protection (PC) was designed to reduce the impact of frost damage on the water conveyance canal. The soil slope was used for the comparison of tests. The different forms of PC and soil slope are shown in Fig. 4.

## Experimental analysis

### Monitoring methods

**Figure 5:**
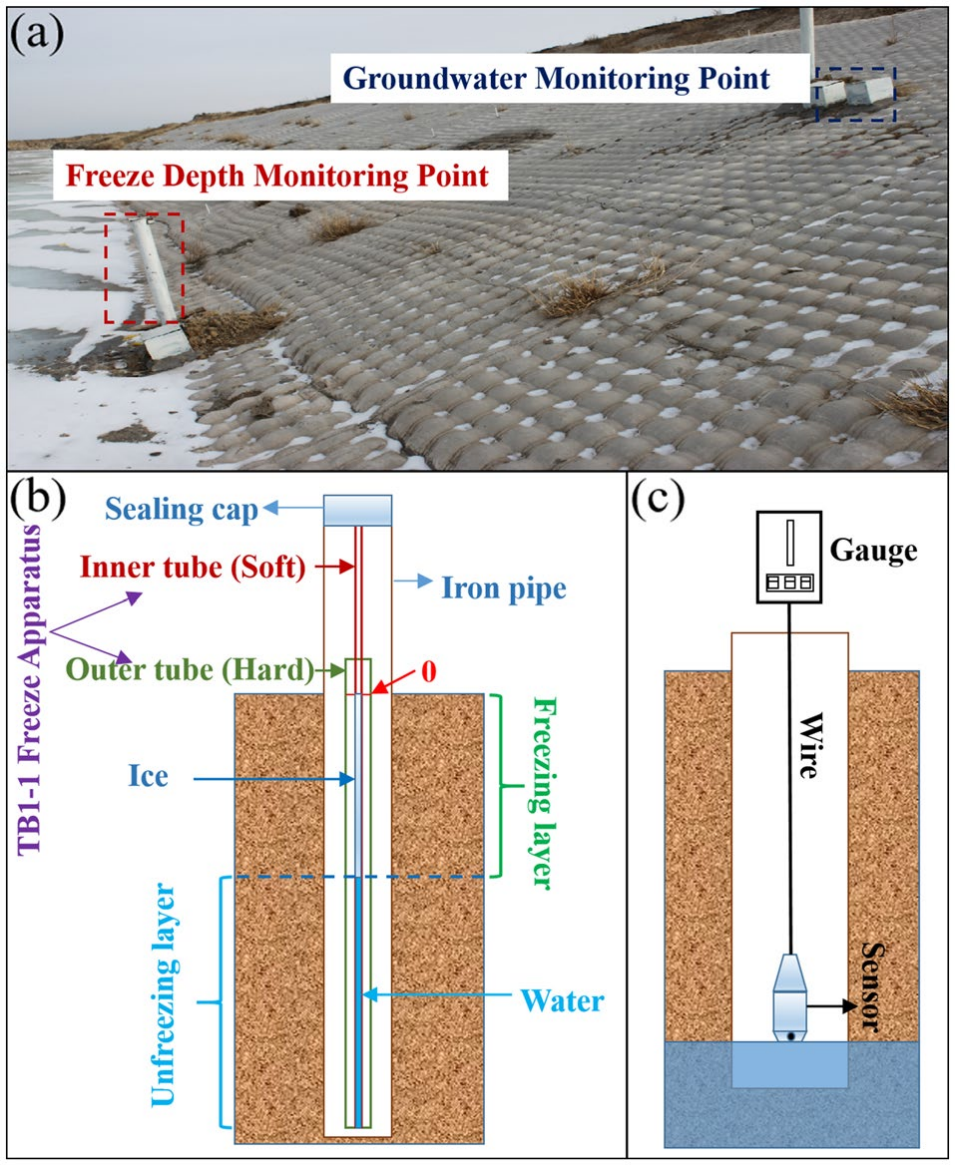
Field tests in 47 km test site ((**a**) freeze depth and groundwater table monitoring in the field, (**b**) freeze depth monitoring diagram, (**c**) groundwater level monitoring diagram).

To determine the characteristics of freeze depth and the effects of groundwater level on the slopes of the canal, 25 m test sections were established at three different locations (hereafter referred to as 2 km test site, 4 km test site and 47 km test site) in the main canal of the Hada mountain water conservancy project. The freeze depth and groundwater level monitoring devices were installed at the top, middle and bottom of the slope as shown in Fig. 5. The freeze depth monitoring devices were TB1-1 Freeze Apparatus (Fig. 5b) buried at the depth of 2.10 m. The TB1-1 Freeze Apparatus consisted of two parts, the inner tubes and the outer tubes. The outer tubes were made of hard rubber tubes marked with a 0 line. The inner tubes were made of soft rubber tubes with centimeter graduations, which were filled with local clean water (river, well or tap water) to the 0 line. The freeze depth of soil was indirectly measured by measuring the length of ice columns in inner tubes. The accuracy of this device was ± 1 cm. Groundwater level was measured by Pressure Hydrometer (Fig. 5c). The sensor of Pressure Hydrometer was put down into the logging well and when the pressure meter reading changed significantly, the length of the logging line was recorded as the groundwater level at this point. The deviation of the sensor was less than 0.2% FS.

### Freeze depth and groundwater changes

**Figure 6:**
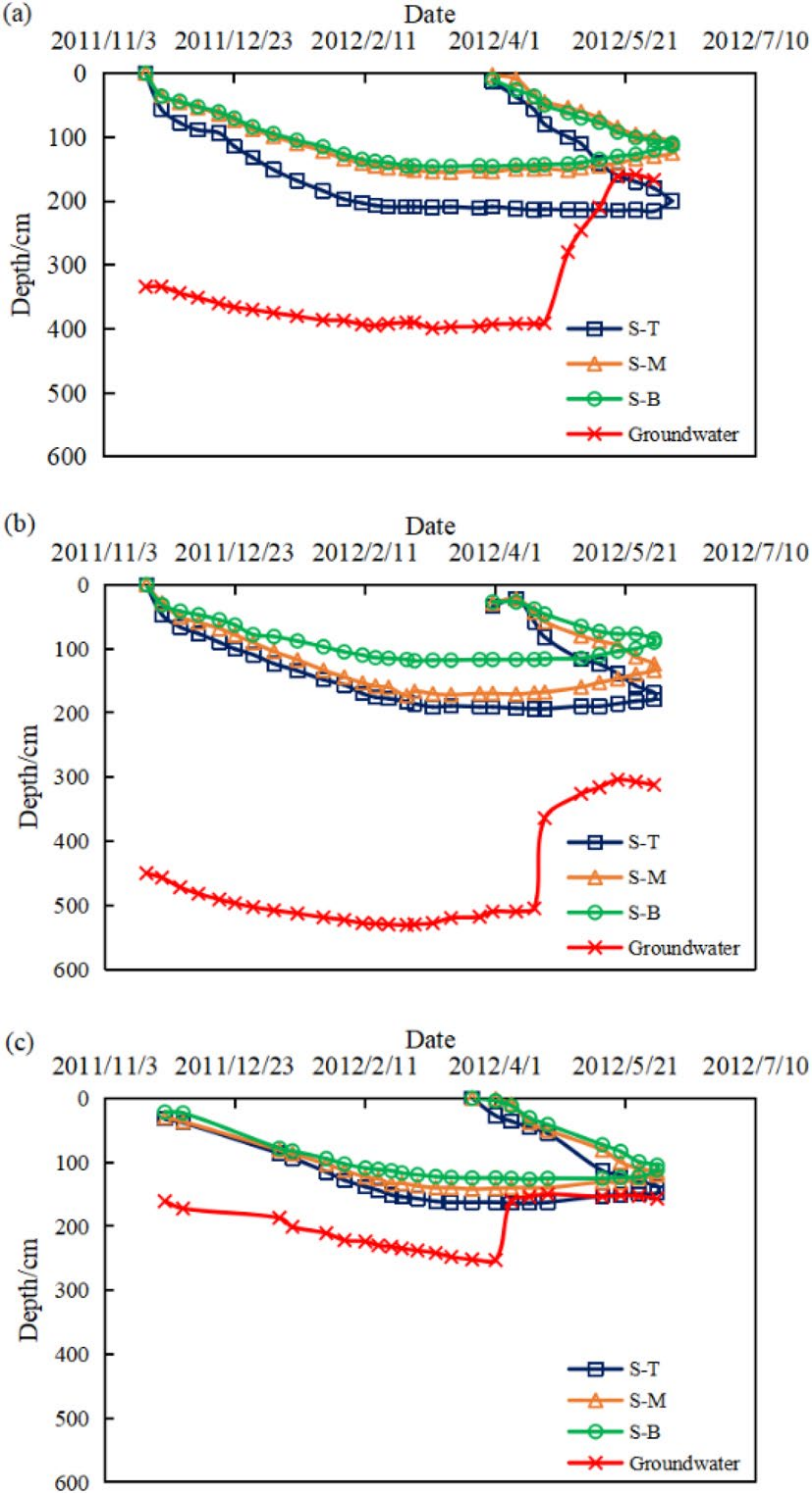
Soil slope—groundwater, freeze depth monitoring curve ((**a**) 2 km test site, (**b**) 4 km test site, (**c**) 47 km test site. *S-T* top of slope, *S-M* middle of slope, *S-B* bottom of slope).

The changes in groundwater level in the region and the freeze–thaw lines on the soil slopes between 2011 and 2012 are shown in Fig. 6. The freezing and thawing curves of the soil and the groundwater curves of the three test sites were similar during the freeze–thaw cycle in 2011–2012, the soil started to freeze at the beginning of November and the freeze depth increased till the mid-February of the year following. The freeze depths in 2 km and 4 km test sites were similar with a maximum freeze depth of about 200 cm at the top, a maximum freeze depth of about 150 cm in the middle and a maximum freeze depth of about 120 cm at the bottom of the slope. The freeze depth of soil in the 47 km test site was less than the previous two test sites with a maximum freeze depth of about 160 cm at the top, 140 cm in the middle and 120 cm at the bottom of the slope. The freezing remained until mid-March when the soil thaws migrated from the surface to the maximum freeze depth. The freezing has almost disappeared by mid-May. The groundwater level declined as the freeze depth increased. The groundwater level remained stable as well when the freeze depth became stable. The groundwater level rose rapidly with the rise in temperature at the start of April due to the release of water from the canal and thawing of soil.

**Figure 7:**
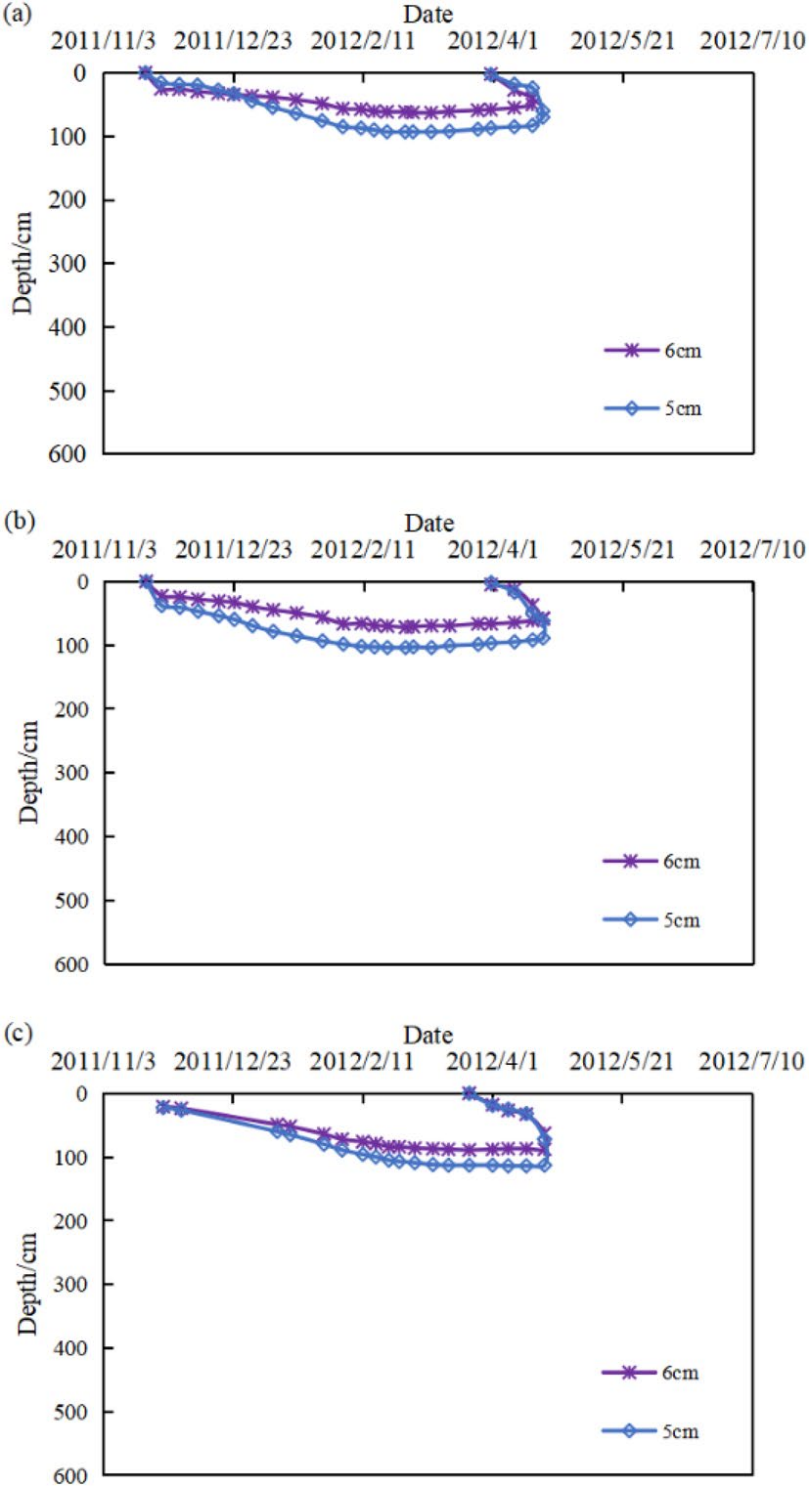
PIB—freeze depth monitoring curve ((**a**) 2 km test site, (**b**) 4 km test site, (**c**) 47 km test site, 6 cm-Slopes protected by 6 cm thick insulation boards, 5 cm-Slopes protected by 5 cm thick insulation boards).

The difference caused by the effects of temperature, wind speed, wind direction and external loads on the freeze depth for the same locations of soil was slight^21^. The main factor contributing to the difference was the effect of water content on the soil. The soil at the bottom of slope had a higher water content than soil at the top. The temperature at the bottom of the slope was relatively higher than that at the top because of the effect of latent heat of phase change in water and ice. Therefore, the freeze depth at the bottom was shallower than that of the top. The freeze depths were measured at the top of the slope when 5 cm and 6 cm Polyurethane insulation boards + Concrete board were used for slope protection. The freeze depths of the soil became shallower as the insulation boards became thicker as shown in Fig. 7. The slope protection structure proposed in this paper could significantly reduce the freeze depth which was about 80–100 cm comparing with the freeze depth in soil slopes. Besides that, the slope thawing was advanced under this type of slope protection, and the freezing of water disappeared in mid-April which was as the freezing of water disappeared almost a month earlier.

### Water content changes in the soil before and after freezing

**Figure 8:**
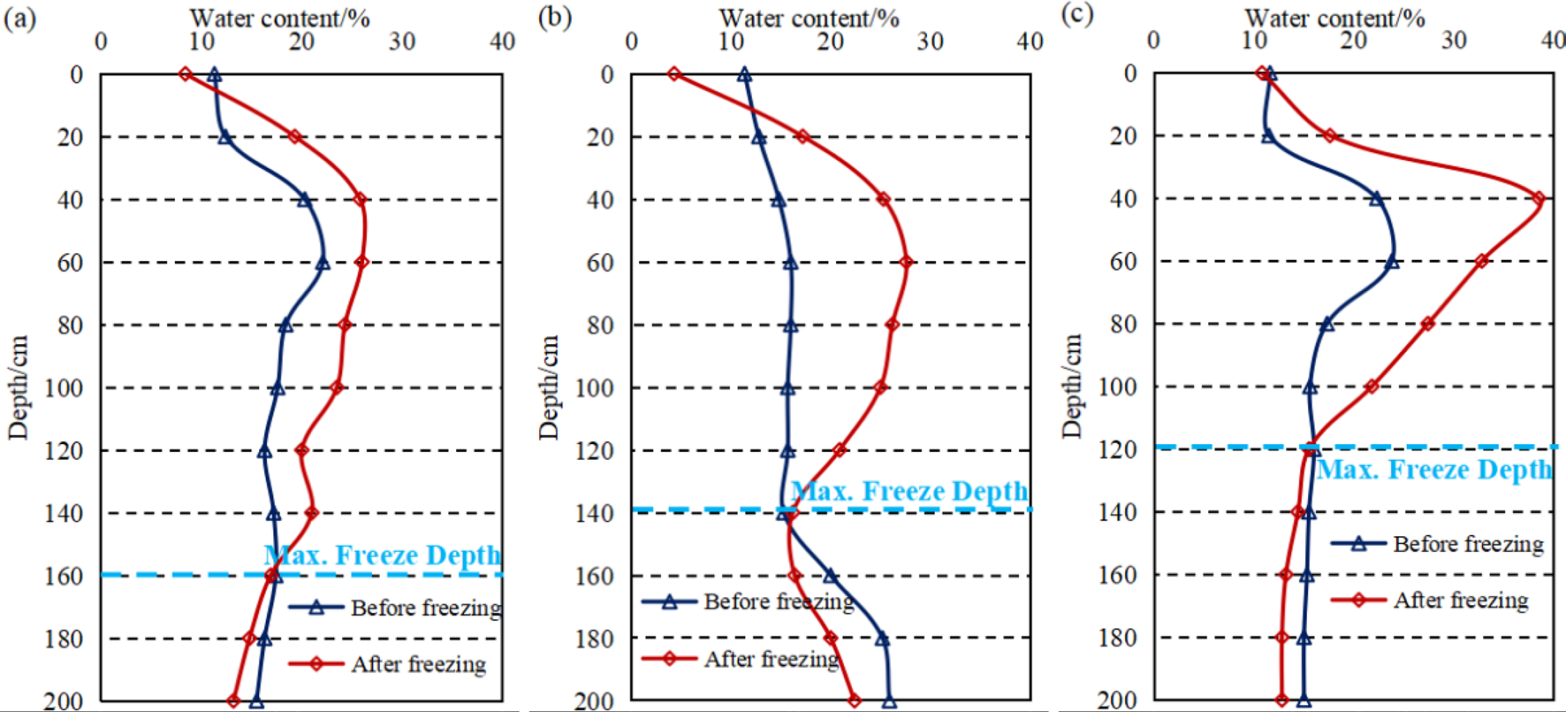
Moisture content change curve of the soil before and after freezing ((**a**) top of slope, (**b**) middle of slope, (**c**) bottom of slope).

The effect of water content on the freeze depth of soil was discussed in “Freeze depth and groundwater changes” section. The water content of soil was measured before and after freezing at different depths of the top, middle and bottom of slope to further verify the conclusions by taking the soil slope of the 47 km test site as an example. The soil water content-depth variation curves are shown in Fig. 8. The water content of surface soil in all three locations of slope was around 10% before freezing in the range of 0–60 cm below the surface, the water content increased with depth as shown in the figure. The high water content of topsoil might be related to the infiltration of surface water. The bottom of slope was close to the water in canal and the water content of soil at bottom was relatively higher than the other two locations due to the action of the flowing water. The water content of topsoil decreased slightly after the water in soil froze which was associated with the sublimation of ice. As the ice front migrated downwards deeper depth, the unfrozen water gradually migrated towards the ice front causing a high-water content of soil than that before freezing. The water content of soil after freezing was lower than that before freezing under the maximum freeze depth due to the migration of water in soil. The water content of soil at the bottom of slope was the highest and the latent heat of phase change was higher among the three test sites, thus the freeze depth at the bottom of slope was less than that at the other two locations of the slope.

## Numerical model for hydrothermal coupling incorporating phase change effects

Seasonal freeze regions are affected by temperature and water freeze in soil causing frost damage. Philip and Devries first proposed the theory of hydrothermal coupling and proposed a nonlinear hydrothermal coupling model based on the principle of viscous fluid flow and heat balance in porous media^22^. Based on this theory, several numerical models of hydrothermal coupling have been proposed, and the problem of hydrothermal coupling of geotechnical engineering in cold regions has been successfully resolved. The study of hydrothermal coupling has been gradually developed and the application of hydrothermal coupling has been increasing recently. The following assumptions are made in the hydrothermal coupling model to improve the efficiency of the calculation^23^:

1. The soil medium is a homogeneous isotropic pore medium consisting of unfrozen water, ice and soil skeleton which does not deform during the freezing process.
2. Water migration in geotechnical medium without the contribution of air to water migration.
3. The latent heat and heat transfer processes of water ice phase change is calculated.

The modelling is carried out by temperature and water fields under the aforementioned assumptions and the different physical field control conditions are as follows:

### Water field equations

The migration of unfrozen water in the soil follows Darcy’s law^24^. The Darcy’s Law interface contains an implementation of Darcy’s law by using the Storage Model node which explicitly includes an option to define the linearized storage *S* (1/Pa) using the compressibility of the fluid and the porous matrix^25^:

3 $$ Q_{{\text{m}}} = \rho S\frac{\partial p}{{\partial t}} + \nabla \cdot \rho \left[ { - \,\frac{\kappa }{\mu }\left( {\nabla p + \rho g\nabla D} \right)} \right], $$

where *Q*_m_ is a mass source representing the additional liquid water due to melting of the ice inclusion; *ρ* is the density of soil (kg/m^3^); *t* is time (s); *p* is the pressure (kPa); $\nabla$ is the Laplace operator; *g* is the acceleration of gravity (m/s^2^); *μ* is the dynamic viscosity (Pa∙s); *κ* is the hydraulic conductivity (m/s); $\nabla {\text{D}}$ is the gravitational potential gradient; *S* is the water transfer model calculated according to Eq. (4) ^25^.

4 $$ S = S_{w} \cdot e \cdot \beta , $$

where *S*_w_ is the saturation of unfrozen water in the soil; *e* is the porosity ratio of the soil, as the water in the soil freezes, the pores are blocked by ice, resulting in a lower porosity ratio, which can be calculated according to Eq. (6); *β* is the effective compression factor, which is the combined value of water, ice and solid matrix compressibility.

The gravity term in Eq. (1) is neglected for simplicity. The variable *Q*_m_ is a mass source representing the additional liquid water due to the melting of ice inclusion^25^:

5 $$ Q_{{\text{m}}} = S_{{\text{w}}} \cdot e(\rho_{i} - \rho_{{\text{w}}} )\frac{{\partial S_{{\text{w}}} }}{\partial t}, $$

where *ρ*_i_, *ρ*_w_ are the densities of ice and water respectively (kg/m^3^).

6 $$ e = e_{0} \cdot S_{w} , $$

where *e*_0_ is the initial porosity ratio.

When considering the ice-water phase change, the saturation of unfrozen water in the soil, *S*_w_ depends on the phase change and can be calculated as shown in Eq. (7) ^25^.

7 $$ S_{{\text{w}}} = S_{{\text{r}}} + (1 - S_{{\text{r}}} ) \cdot \theta_{2} , $$

8 $$ \theta_{1} + \theta_{2} = 1, $$

where *S*_r_ is the residual liquid water saturation; *θ*_1_, *θ*_2_ is smooth step function defined in the phase change material node, the step function *θ*_2_ (T) is zero for temperature below the melting temperature; *T*_pc_, and is equal to 1 for temperature above *T*_pc_. It is assumed that the mushy ice zone extends from 0 to − 1 °C in the interfrost benchmark. Therefore, *T*_pc_ is set to − 0.5 °C and the transition interval of *θ*_2_ is defined as 1 K.

The ice clogs porosity in the soil leading to a reduction in the porosity ratio considering the ice-water phase change which leads to a lower permeability of the soil. The *k* expressed in terms of saturation *S*_w_ can be calculated by Eq. (9) ^25^.

9 $$ k = k_{s} \cdot 10^{{ - I \cdot e \cdot (1 - S_{{\text{w}}} )}} , $$

where *k*_s_ is the coefficient of permeability of saturated soil (m/s) and *I* is the impedance factor.

### Temperature field equations

The differential equation for heat conduction in frozen soil is^25,26^:

10 $$ (\rho C)_{{{\text{eq}}}} \frac{\partial T}{{\partial t}} + \rho C_{{\text{w}}} u \cdot \nabla T + \nabla \cdot ( - k_{{{\text{eq}}}} \nabla T) = Q, $$

where (*ρC*)_eq_ is the effective volumetric heat capacity at constant pressure; *T* is the temperature of the soil at different moments (°C); *k*_eq_ is the effective thermal conductivity (W/m·K); *Q* is a heat source (W/m^3^); *C*_w_ is the effective fluid’s heat capacity at constant pressure; ***u*** is the velocity field, either an analytic expression or computed from a Fluid Flow interface. It should be interpreted as the Darcy velocity i.e., the volume flow rate per unit cross sectional area.

### Model validation

**Figure 9:**
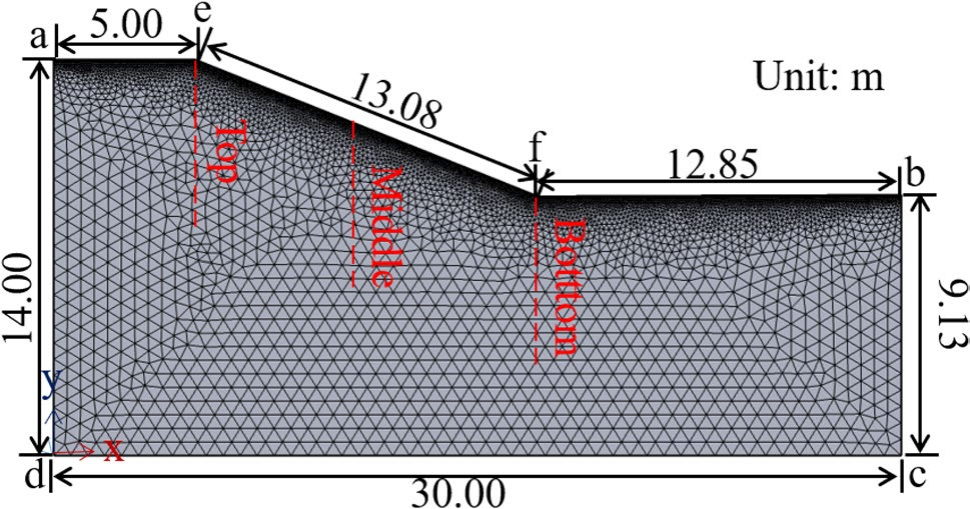
Numerical calculation model.

A numerical model was constructed based on the 47 km test section among the three test sites of this paper to simplify the calculations. The boundary conditions of the numerical simulation calculation model and the dimensions of each part were shown in Fig. 9. The model contained 5544 grid vertices and 10,521 elements. In the temperature field, it was assumed that heat exchange occurred at the surface and the rest of the boundaries were set as the thermal insulation boundaries^21,24^. The temperature boundary condition as shown in Eq. (2) was imposed with an initial value of 15 °C. The external water recharge to the soil has been neglected in this paper as there was no drainage in the water field of conveyance canal in winter. Therefore, the soil was considered as unsaturated and the boundaries were set as zero flux boundaries^21,24^. The boundary conditions and specific material parameters were shown in Tables 1, 2 and 3. The transient calculation method was used to calculate the freeze depth and the change in water content of the soil over 365 days. The freeze depth, as well as the change in water content, were monitored at the three locations marked by the dotted lines.

**Table 1:** Boundary conditions.

Line	Temperature field	Water field
a–e–f–b	Temperature boundary	Zero flux boundary
a–d	Thermal insulation boundary	Zero flux boundary
b–c	Thermal insulation boundary	Zero flux boundary
c–d	Thermal insulation boundary	Zero flux boundary

**Table 2:** Material properties.

Material	*ρ* (kg/m^3^)	*C* (J/kg·°C)	*λ* (W/m °C)
Clay	1910	1460	2.50
Water	1000	4200	0.63
Ice	918	2100	2.31

**Table 3:** Parameters for seepage models of unsaturated soils.

Parameters	*I*	*e*_0_	*μ* (Pa·s)	*w*_sat_	*S*_w_	*S*_r_	*k*(m/s)
Clay	50	0.3	1.793e−3	0.35	0.68	0.14	9.62 × 10^–7^

**Figure 10:**
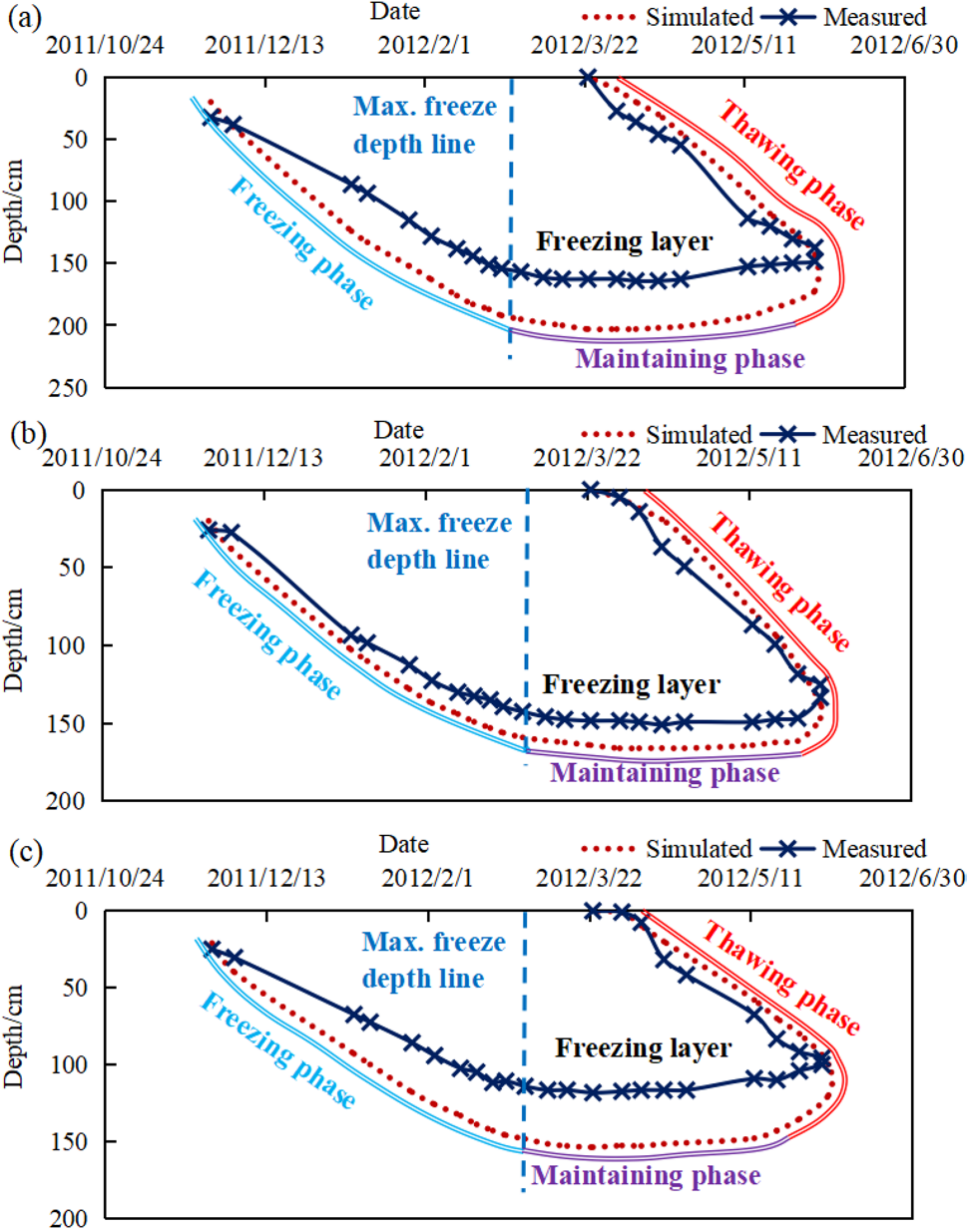
Freeze–thaw curves for slopes ((**a**) Top of slope, (**b**) Middle, (**c**) Bottom slope).

The measured and simulated values of freeze–thaw process at the top, middle and bottom of slope in a single freeze–thaw cycle are shown in Fig. 10. Although there was a deviation between the simulated and measured values with a maximum deviation of about 30 cm. The observed difference was mainly caused by the fact that the temperature boundary conditions in numerical calculation were fitted to the measured results. The actual temperature boundary conditions in the field were influenced by solar radiation, wind direction and speed as well as rainfall making it impossible to fully introduce the computational model. However, the results of the simulations still reflected the freeze–thaw process on the slopes. It was clear by comparing the results that the freezing process manifested three main stages during a freeze–thaw cycle.

1. *Freeze developing phase* During this phase, the ice front developed downwards due to the decrease in temperature leading to an increase in the freeze depth.
2. *Freeze maintenance phase* When the ice front surface developed to a certain depth, the ice front neither retreated nor continued to develop and remained stable.
3. *Thawing phase* In this phase, the temperature warmed up to greater than 0 °C and the frozen soil began to thaw until the soil was no longer frozen.

The distribution characteristics and change processes between measured and simulated freezing/thawing depths were similar. The numerical results showed that the numerical model developed in this paper for calculating the slope of water conveyance canal was reliable and could be applied to the analyses of temperature-freeze depth-water content.

### Temperature-freeze depth-water content regime

**Figure 11:**
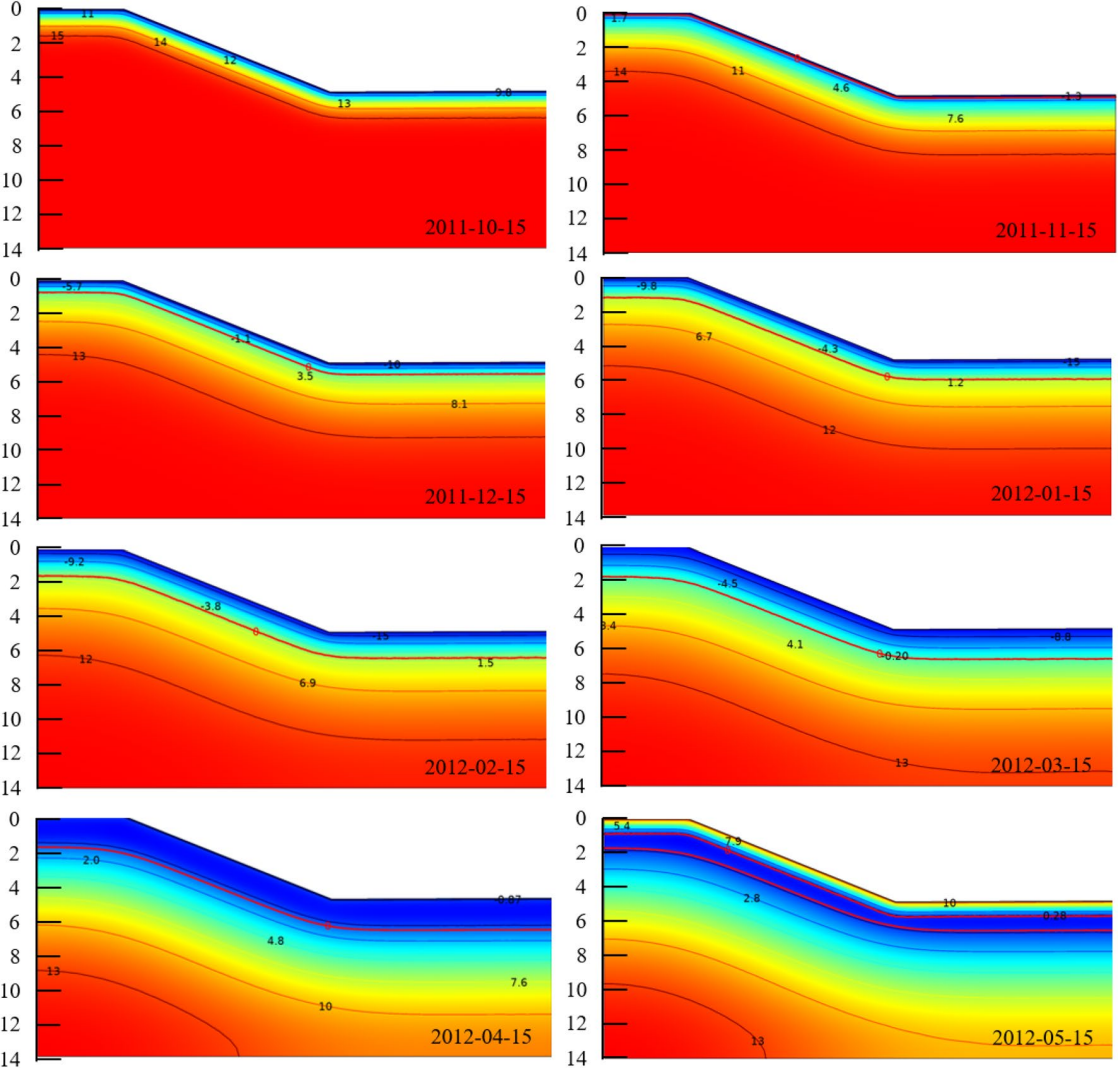
Temperature field distribution at different times (unit: °C).

**Figure 12:**
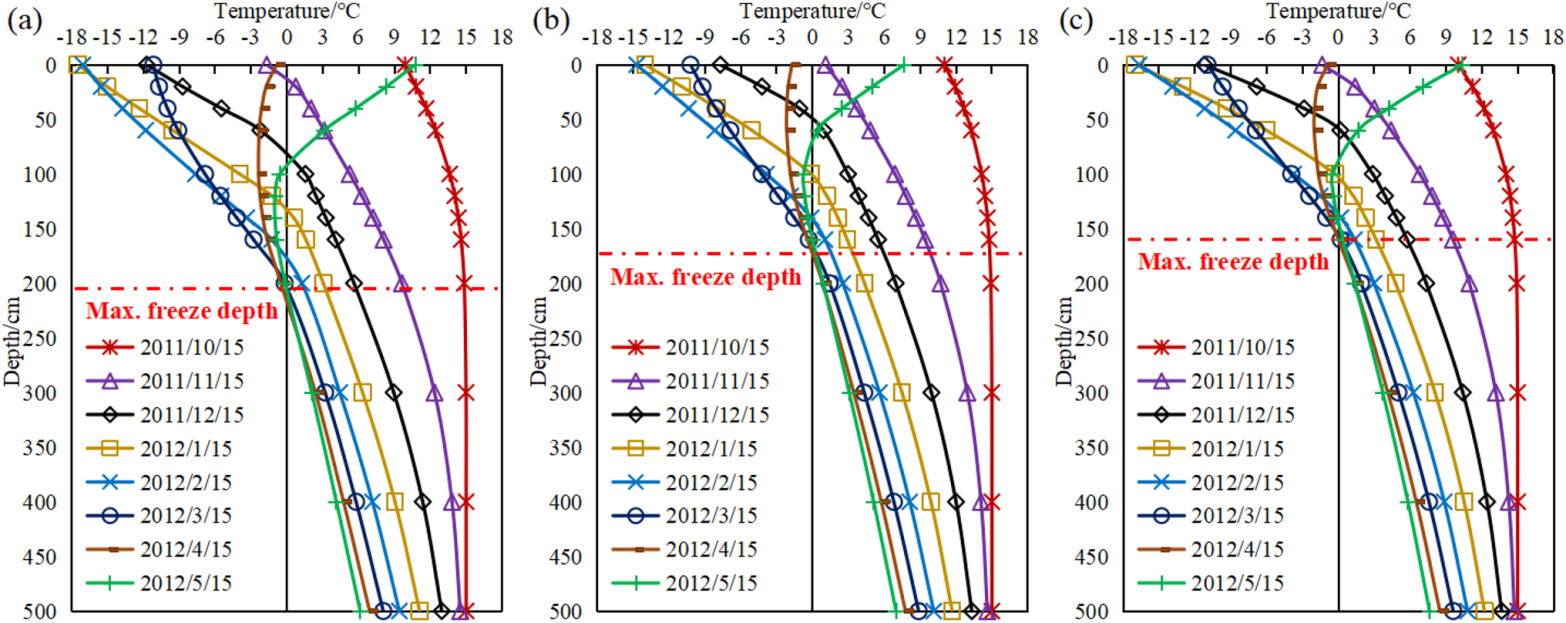
Temperature variation curves ((**a**) top of slope, (**b**) middle of slope, (**c**) bottom of slope).

The main factor influencing water migration was the coupling of temperature and water fields during soil freezing and thawing i.e., the movement of water under the effect of temperature gradients^21^. The temperature distribution of the numerical simulation is shown in Figs. 11 and 12 with the initial temperature field set at 15 °C in mid-October. The water freezing occurred around mid-November 2011 and the freeze depth gradually increased with the decrease in temperature. Water freeze reached the maximum freeze depth in mid-March 2012 and remained stable. Comparing the temperature variation curves at different depths for the three locations, the freeze depths were about 200 cm, 160 cm and 150 cm from top to bottom of the slope.

**Figure 13:**
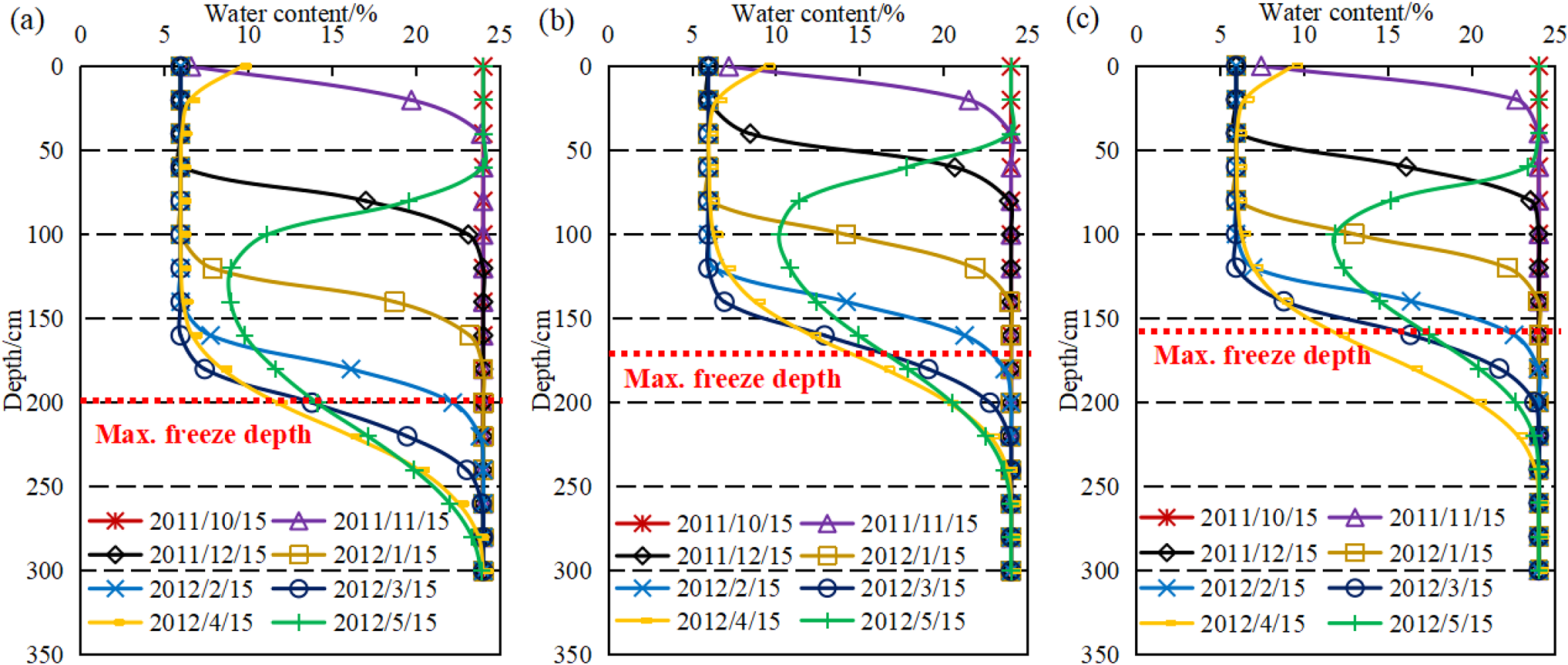
Water content variation curves ((a) top of slope, (**b**) middle of slope, (**c**) bottom of slope).

The water in pores gradually froze and the water content decreased as the temperature decreased. The change in water content during the conversion of water to ice is shown in Fig. 13. The water content was 24% on October 15th 2011 as shown in the figure. In November, the water in soil froze to a depth of around 50 cm. At this time, the soil below 50 cm has not frozen and the water content remained at the initial water content. The water content remained stable at the same time after the freeze depth remained stable in mid-March. The ice in soil about 20 cm below the surface gradually thawed and melted when the temperature warmed up in April and the water content returned to its initial value. The curves of water content showed that after the water froze, the water content of soil below the maximum freeze depth also decreased. It was not until the freeze has completely melted that the water content gradually returned to its initial value, which was inextricably related to the water migration during the freezing process.

### Anti-frost structures on the slopes of water conveyance canals

**Figure 14:**
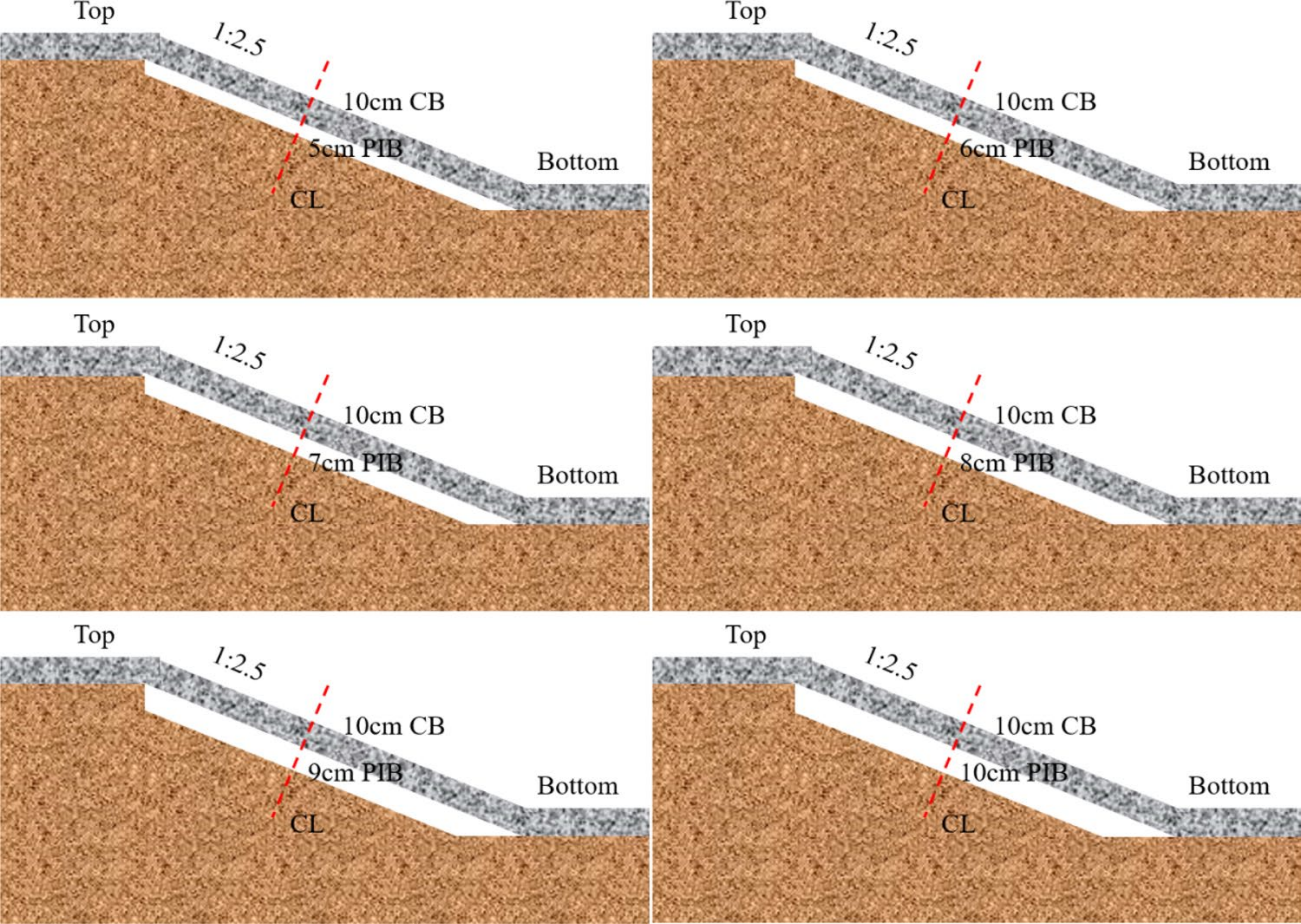
Anti-frost structures on the slopes of water conveyance canals in seasonal frozen regions (*CB* concrete board, *PIB* polyurethane insulation board, *CL* low liquid limit clay).

Water conveyance canal projects in seasonal freeze regions are often damaged by the freeze–thaw action of the soil. Therefore, anti-frost structures should be adopted to eliminate or reduce the soil freeze, which can effectively reduce the risk of damage to the slope. According to the measured and simulated values in the previous sections, the degree of freeze was mainly influenced by temperature as well as water content. Therefore, the insulation boards were most commonly used for thermal insulation and water insulation. According to the results of anti-frost tests on insulation boards in the test areas, the small thermal conductivity of polyurethane material enabled the heat balance between heat absorption and heat release, thus resulting in the subsoil not freezing. For further determination of insulation board thickness, a structural form (Polyurethane insulation board + Concrete board) as shown in Fig. 14 was used for anti-frost simulation.

The maximum freeze depth distribution could be computed based on numerical model established in this paper. A thin layer boundary condition was used instead of the insulation board solid mesh for the convenience of modelling. The insulation board, as well as concrete material parameters, are shown in Table 4.

**Table 4:** Parameters of insolation boards and concrete boards.

Material	*λ*·(W/m·°C)	*ρ* (kg/m^3^)	*C*·(J/kg °C)
Concrete	1.800	2500	880.0
PIB	0.026	48	1330.0

**Figure 15:**
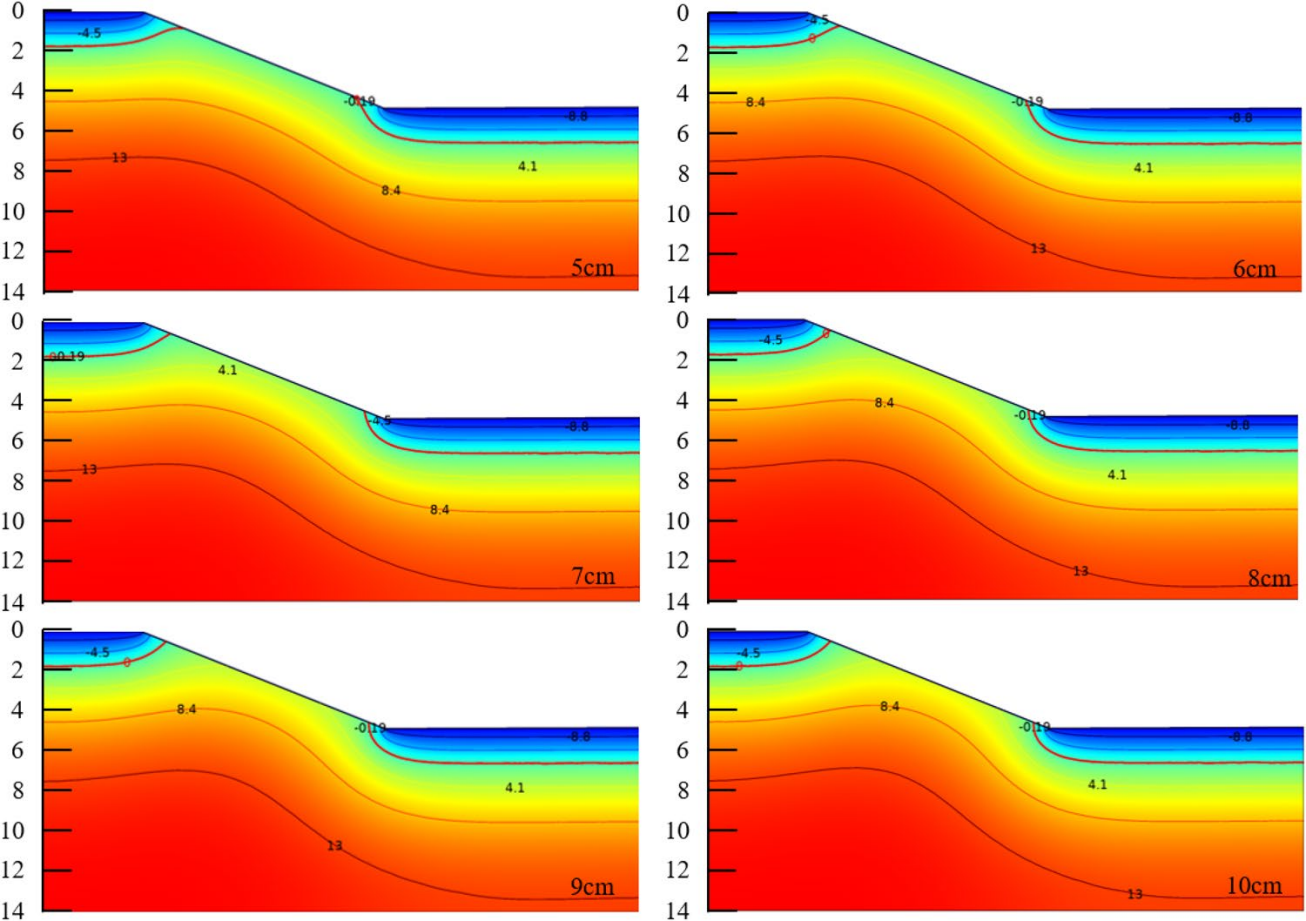
Temperature fields of the six anti-frost structures at maximum frost depth time (5–10 cm is thickness of Polyurethane insulation board).

The results of the temperature field simulations are shown in Fig. 15, where the 0 °C isotherms are used as the maximum freeze depth. The changes in freeze depth and water content at three locations are shown in Table 5.

**Table 5:** The max. frost-depth and water-content for the six anti-frost structures.

PIB/cm	T		M		B	
	Freeze depth/cm	Water content/%	Freeze depth/cm	Water content/%	Freeze depth/cm	Water content/%
0	200	11.3	160	11.3	150	14.6
5	141	14.7	0	24.0	113	14.7
6	138	14.8	0	24.0	111	14.8
7	135	14.9	0	24.0	108	14.9
8	133	14.9	0	24.0	106	14.7
9	130	14.6	0	24.0	104	14.7
10	129	14.9	0	24.0	103	14.8

Comparing the effect insulation board of different thicknesses on anti-frost, it showed that 5 cm and 6 cm insulation boards could significantly reduce the freeze depth. The effect of increasing the thickness of insulation boards on reducing the freeze depth did not change significantly. In actual engineering applications, increasing the thickness of the insulation boards is undoubtedly increase the cost of project, but the effect of improving insulation boards is not obvious. According to presented researches, the insulation boards with thicknesses of 5 cm and 6 cm could improve the anti-frost capacity of the slope. Regions with serious frost damage can refer to the research results of this paper and can appropriately increase the thickness of the insulation boards. Comparing the water content at maximum freeze depth showed that at the maximum freeze depth of the unprotected slope, the water content of the soil remained 11%, i.e. most of the water in soil was frozen. However, although the water content of soil with protective structures decreased, the unfrozen water content of the soil remained around 14.8%. The freezing action reduced and water at the maximum freeze depth cannot freeze sufficiently with the protection of the insulation boards.

Under the protection of polyurethane insulation boards in the middle of the slope, the freeze depth was almost negligible and differed considerably from the actual situation. This was due to the simplification of boundary conditions in numerical calculations and the fact that external factors (solar radiation, wind speed, wind direction and precipitation, etc.) were not considered in the calculation process. Therefore, the results obtained differed considerably from reality, but they still illustrated the usability of polyurethane boards in the design of anti-frost structures.

## Discussion

### Characteristics of temperature and freeze depth change in seasonal freeze regions

**Figure 16:**
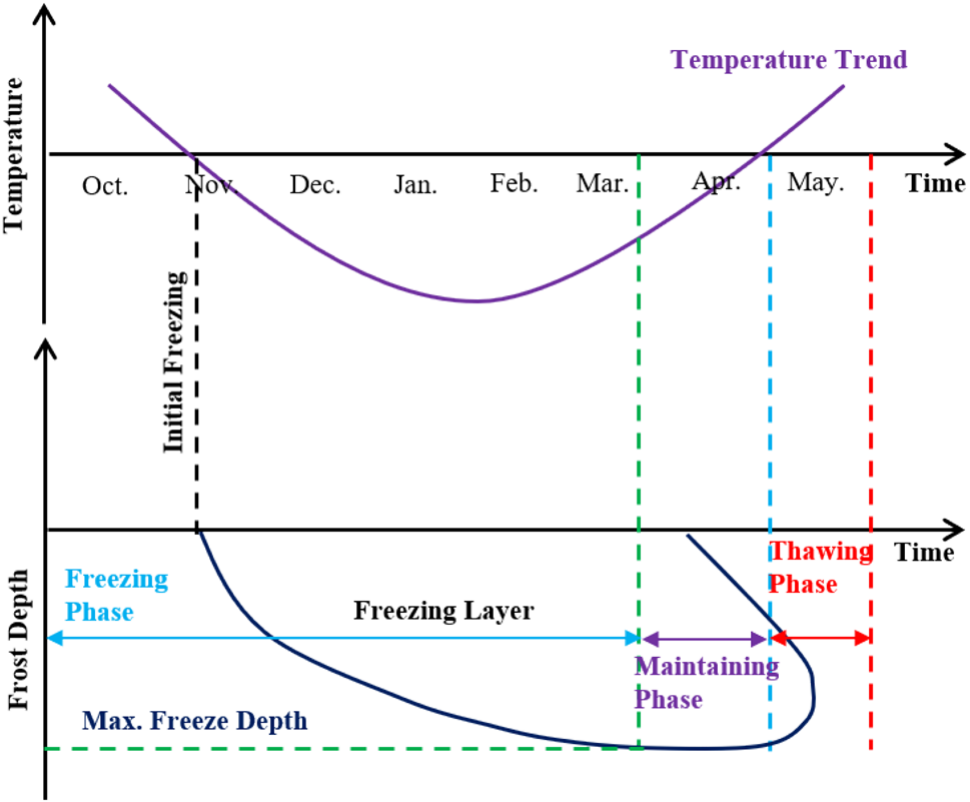
Freeze depth-time curve.

In seasonal freeze regions, the water in soil freezes under the cold temperature^27–29^. The temperature changes dramatically from the ground surface to the maximum freeze depth—a process that directly affects the direction and intensity of water migration^21^. In this paper, the trend of soil freeze depth is summarized in Fig. 16 by field tests as well as numerical simulations. The temperature gradually drops from October to November each year as shown in the figure causing the water in soil to freeze. Thereafter, the freeze depth has increased when it reaches the maximum freeze depth until March. The temperature increases to above zero and the soil melts in both directions from the surface and the maximum freeze depth after the maximum freeze depth remains until April and the freezing of soil generally disappears by the end of May.

### Characteristics of temperature and water variation in seasonal freeze regions

The temperature at the surface in seasonal freeze regions varies widely depending on the seasons, resulting in differences in temperature from the surface to the maximum freeze depth^21,30^. For example, at different locations on the slopes, the freeze depth varies between shaded and sunny slopes^31,32^.

**Figure 17:**
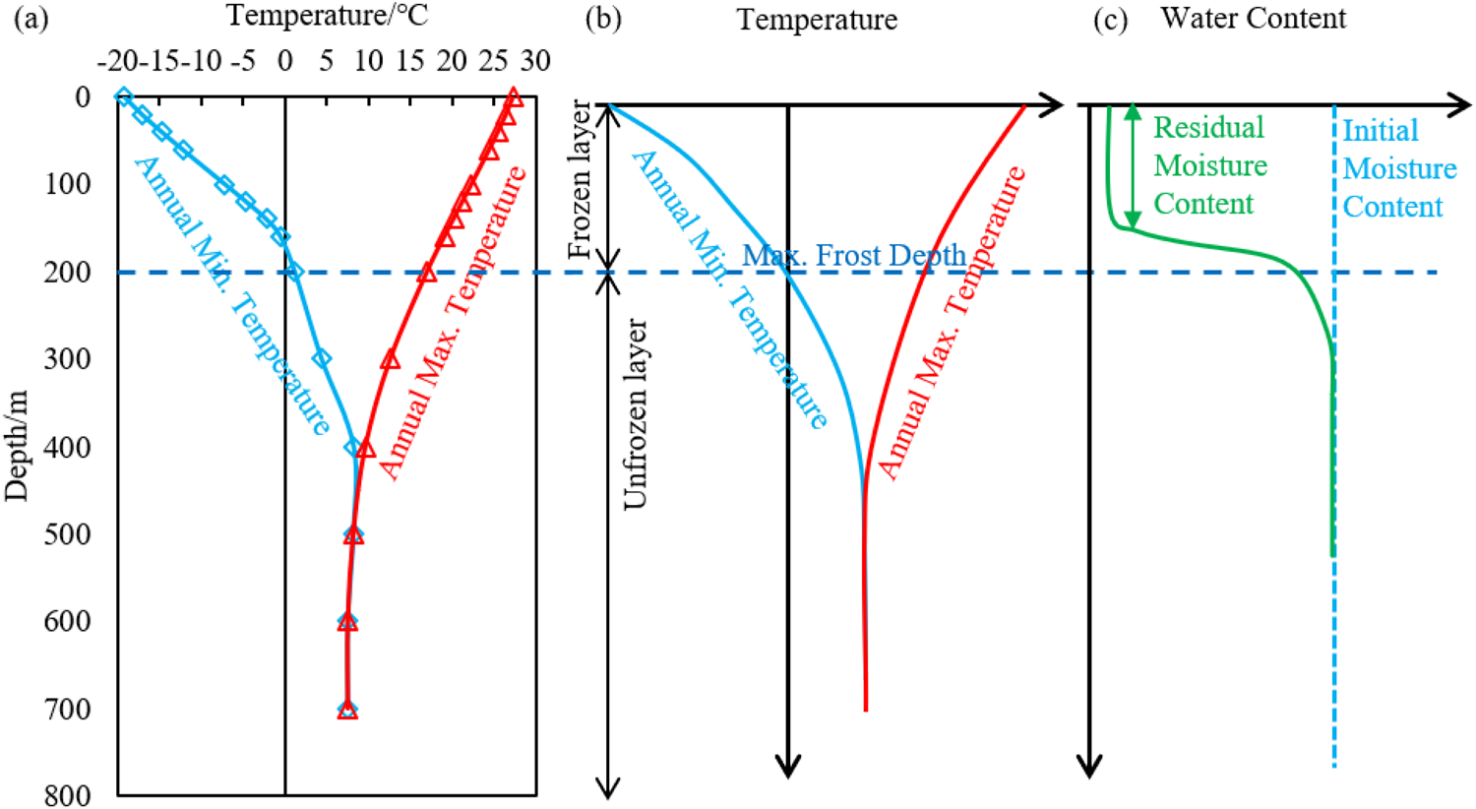
Characteristics of temperature-depth-water content variation in the seasonal frozen regions ((**a**) simulated value, (**b**) temperature-depth trend, (**c**) water content-depth trend).

The freeze depth also varies as shown in Fig. 17. The water in soil gradually freezes as the temperature decreases from the surface to the maximum freeze depth (Fig. 17a), this part of the soil layer is called the frozen layer and below the frozen layer is the unfrozen layer. The freeze depth affects the water content of soil (Fig. 17c). In the simulations of this paper, the water storage model is applied in Comsol to fully investigate the effect of freezing water on the soil porosity ratio as well as the permeability coefficient, and the results are more reflective of the hydrothermal coupling during the freezing and thawing process. The water content of soil in the frozen layer decreases to the residual water content. Although freezing does not occur below the maximum freeze depth, the water content of the soil under the maximum freeze depth also decreases. This is because the ice front is constantly developing downwards during the freezing process and water migration occurs under the influence of temperature as well as soil pore capillary forces, resulting in a decrease in the water content of the soil below the maximum freeze depth. The water migration occurs by gravity and the water content of the soil gradually converges to its original state after the soil has completely melted.

## Conclusions

This paper investigated the characteristics of temperature-freeze depth-water content of slope based on the combination of field tests and numerical simulations, and compared the effect of different thicknesses of polyurethane materials, the following conclusions are drawn:

1. The ice front gradually developed downwards as the temperature decreased, causing a decrease in the path of water migration which was beneficial for water migration. The water content of soil below the maximum freeze depth was less than the initial water content after the water froze to the maximum freeze depth which was inseparably related to water migration.
2. The freeze depth of soil decreased significantly when polyurethane material was used for anti-frost protection, which was beneficial for the safety of canals. Comparing the anti-freeze effect of different thicknesses of insulation boards, it could be concluded that 5–6 cm thick insulation boards could significantly reduce the freeze depth by approximately 60 cm. The anti-freeze effect did not increase obviously when the insulation board was thicker than 6 cm.
3. The thickness and temperature distribution of the frozen layer in seasonal frozen soil areas were the important factors influencing the damage of water conveyance canals. The polyurethane insulation boards precisely met the criteria to fulfil the requirements of increasing the heat entering into slope or reducing the heat diffusion of soil and reducing the freezing. Therefore, such materials have broad prospects for application in the prevention of freezing.

The results presented in this paper has provided important technical guidance for the implementation of frost prevention measures and the optimization design of an anti-frost structure on canals in freeze regions and can be applied to the design of water conveyance in seasonal freeze regions to determine proper insulation board thickness.
